# Ceftazidime Is the Key Diversification and Selection Driver of VIM-Type Carbapenemases

**DOI:** 10.1128/mBio.02109-17

**Published:** 2018-05-08

**Authors:** Laura Martínez-García, José M. González-Alba, Fernando Baquero, Rafael Cantón, Juan Carlos Galán

**Affiliations:** a Servicio de Microbiología, Hospital Universitario Ramón y Cajal, Madrid, Spain; b Instituto Ramón y Cajal de Investigación Sanitaria (IRYCIS), Madrid, Spain; c Red Española de Investigación en Patología Infecciosa (REIPI), Madrid, Spain; d CIBER en Epidemiología y Salud Pública (CIBERESP), Madrid, Spain; e Unidad de Resistencia a Antibióticos y Virulencia Bacteriana, Madrid, Spain; International Health Management Associates, Inc.; Indiana University Bloomington

**Keywords:** VIM, ceftazidime, diversification, evolution, phylogeny

## Abstract

In recent decades, carbapenems have been considered the last line of antibiotic therapy for Gram-negative bacterial infections. Unfortunately, strains carrying a high diversity of β-lactamases able to hydrolyze carbapenems have emerged in the clinical setting. Among them, VIM β-lactamases have diversified in a bloom of variants. The evolutionary reconstructions performed in this work revealed that, at the end of the 1980s, two independent events involving diversification from VIM-2 and VIM-4 produced at least 25 VIM variants. Later, a third event involving diversification from VIM-1 occurred in the mid-1990s. In a second approach to understanding the emergence of VIM carbapenemases, 44 mutants derived from VIM-2 and VIM-4 were obtained by site-directed mutagenesis based on those positions predicted to be under positive selection. These variants were expressed in an isogenic context. The more-evolved variants yielded increased levels of hydrolytic efficiency toward ceftazidime to a higher degree than toward carbapenems. In fact, an antagonist effect was frequently observed. These results led us to develop an experimental-evolution step. When Escherichia coli strains carrying VIM-2 or VIM-4 were submitted to serial passages at increasing concentrations of carbapenems or ceftazidime, more-efficient new variants (such as VIM-11 and VIM-1, with N165S [bearing a change from N to S at position 165] and R228S mutations, respectively) were only obtained when ceftazidime was present. Therefore, the observed effect of ceftazidime in the diversification and selection of VIM variants might help to explain the recent bloom of carbapenemase diversity, and it also represents another example of the potential universal effect exerted by ceftazidime in the selection of more-efficient β-lactamase variants, as in TEM, CTX-M, or KPC enzymes.

## IMPORTANCE

One of the objectives recently proposed by the World Health Organization (WHO) Assembly in the global plan on antimicrobial resistance was to improve the understanding and knowledge of antimicrobial resistance. In the present work, we paid attention to the drivers of diversification and selection of new carbapenemases in Gram-negative bacteria, which occupy one of the most critical places in the WHO priority list of antibiotic-resistant microorganisms. Based on evolutionary-reconstruction, site-directed-mutagenesis, and experimental-evolution approaches, we proposed a critical role of ceftazidime exposure in the selection of VIM carbapenemase variants. This surprising finding is also applicable to other β-lactamases, indicating that ceftazidime, and not other antibiotics, might have a universal effect in the diversification of β-lactamases. Our results might help to define future strategies to reconsider the extended use of ceftazidime.

## INTRODUCTION

For years, carbapenem antibiotics have been considered the last resort for treatment of infections due to multidrug-resistant bacteria, especially in critically ill patients (1, 2). As a consequence of the global spread of bacteria carrying antibiotic resistance genes conferring an extended-spectrum β-lactamase (ESBL) phenotype, such as CTX-M enzymes, the worldwide consumption of carbapenems drastically increased in the period from 2000 to 2010 (3). As a side effect, the proportion of carbapenem-resistant strains (CRS) gradually increased (4). Nowadays, the threat of multidrug-resistant microorganisms, including those resistant to carbapenems, is an international public health crisis (5–8). Aware of this threat, in 2017 the World Health Organization (WHO) classified carbapenem-resistant *Acinetobacter*, *Pseudomonas*, and *Enterobacteriaceae* strains as the most critical group in a list of multidrug-resistant bacteria for urgent attention (http://www.who.int/medicines/publications/global-priority-list-antibiotic-resistant-bacteria/en/). Among CRS, the most widespread resistance mechanism is the presence of β-lactamases with the ability to hydrolyze carbapenems, the so-called carbapenemases. These enzymes are grouped in three molecular classes: class A enzymes (serine-β-lactamases) like KPC, class B enzymes (metallo-β-lactamases [MBLs]) like VIM and NDM, and class D enzymes (oxacillinases) like OXA-48 (9). Excluding the chromosomal carbapenemase genes, with low rates of spreading from their original species, carbapenemases carried in mobile elements have been increasingly frequently described in the last 2 decades. MBL types were mostly detected in the period between 1997 and 2012 (except for IMP) and serine carbapenemases between 1996 and 2000 (except for KPC and GES), while the detection of OXA-type carbapenemases, such as OXA-23, occurred at the same time as when imipenem was approved for general use (10, 11). However, and contrary to an apparently obvious conclusion, several authors suggest that the clinical use of carbapenems was not responsible for the evolution of certain carbapenemases (10, 11). In class D enzymes, phylogenetic reconstructions showed that new recognized variants are ancestral and probably were present in bacteria before the commercialization of imipenem. On the other hand, among the most prevalent class A and B carbapenemases (KPC and VIM), the description of new variants has been a recent phenomenon, suggesting either environmental pressure for the selection of more-efficient variants or more-intensive CRS surveillance programs or both.

Our group previously described the selective effect of the oxyimino-cephalosporins in the diversification of CTX-M β-lactamases (12). Other authors obtained similar results focusing on TEM extended-spectrum β-lactamases (13). Therefore, it can be suspected that the emergence of new carbapenemase variants must be related to the selection of enzymes with better carbapenem-hydrolyzing activity and, thus, dependent on the level of carbapenem exposure. However, in natural variants of KPC-2, the increases in enzymatic activity are higher against ceftazidime (14) than against carbapenems, and VIM-1 yielded higher efficiencies in hydrolysis of ceftazidime and cefepime than its putative ancestor VIM-4 (15), without relevant changes in activity against the carbapenems. The main objectives of this study are to analyze the selector antibiotics responsible for the explosive diversification of VIM carbapenemases and, in particular, to investigate the role of the oxyimino-cephalosporins from an integrated perspective, including genetics, bioinformatics, and evolutionary biology. Finally, we address whether the effect of oxyimino-cephalosporins, especially ceftazidime, in the recent diversification of β-lactamases could constitute a universal phenomenon.

## RESULTS

### Phylogenetic analysis.

**FIG 1:**
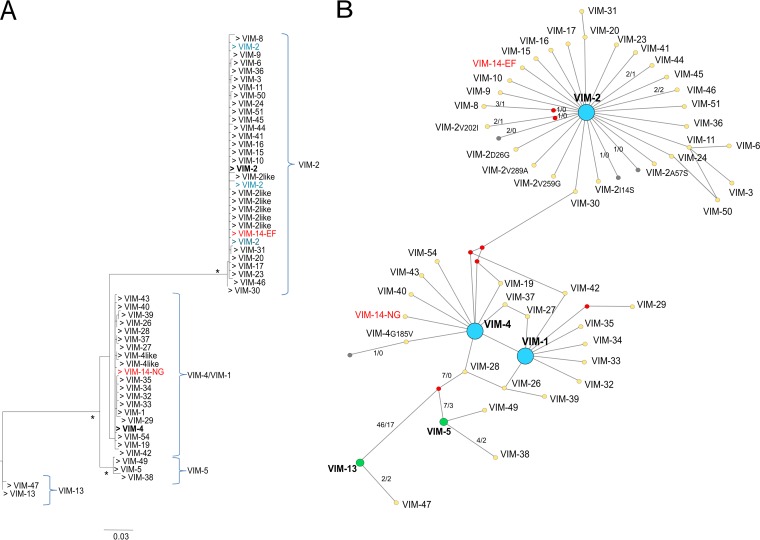
Maximum-likelihood (ML) and network phylogenetic trees of *bla*_VIM_ sequences. (A) ML phylogenetic tree constructed using the PhyML 3.0 program. All sequences except those with gaps and the recombinant ones were used (57 of 438). Support for the relevant nodes was estimated by bootstrapping (1,000 replicates), and only support values of >90% were considered statistically significant (indicated with an asterisk). Four different phylogroups were differentiated, corresponding to VIM-13, VIM-5, VIM-4/VIM-1, and VIM-2. A group was assigned only if there was more than one sequence. The sequences used as reference sequences in the VIM-2 and VIM-4 clusters are marked in boldface. VIM-2-like and VIM-4-like refer to VIM-2 or VIM-4 sequences with 1 amino acid change from the VIM-2/VIM-4 sequences used as the reference sequences. Those highlighted in blue refer to sequences carrying single-nucleotide polymorphisms (SNPs) relative to the sequence used as the reference. An incongruent finding was detected corresponding to two different accession numbers for *bla*_VIM-14_ (EF055455 and NG050341) and allocated to two different phylogroups (VIM-2 and VIM-4 clusters, respectively) (highlighted in red in both panel A and panel B). (B) Phylogenetic network constructed using the Network 5.0.0.1 (Fluxux Technology) software. The dot colors indicate the following: blue dots show the three main diversifications (VIM-2, VIM-4, and VIM-1), green dots show other events of diversification, red dots show the hypothetical ancestors, and gray dots show the VIM sequences carrying SNPs without amino acid changes relative to the sequence of the ancestor. Numbers on a line between an ancestor and a derivative variant indicate the number of nucleotide changes/number of amino acid changes. If no numbers are given, there was a 1/1 change. GenBank accession numbers are shown in Text S1 in the supplemental material.

Four VIM phylogroups could be differentiated according to the results of the maximum-likelihood (ML) tree obtained using the nucleotide sequences (Fig. 1A). The first was formed by two members (*bla*_VIM-13_ and *bla*_VIM-47_), the second corresponded with *bla*_VIM-5_ (including *bla*_VIM-5, -38_, and _-49_), the third was the *bla*_VIM-4_/*bla*_VIM-1_ group, formed by 18 members (*bla*_VIM-4, -1, -14, -19, -26, -27, -28, -29,__-32,-33, -34, -35, -37, -39, -40, -42, -43,_ and _-54_), and the fourth corresponded to the *bla*_VIM-2_ group, formed by 22 members (*bla*_VIM-2, -3, -6, -8, -9, -10, -15, -16, -17, -18, -20, -23, -24, -30, -31, -36, -41,__-44, -45, -46, -50,_ and _-51_). Among all VIM variants analyzed, two recombinant variants were detected, *bla*_VIM-12_ (resulting from a recombination event between *bla*_VIM-1_ and *bla*_VIM-2_) and *bla*_VIM-25_ (resulting from a recombination event between *bla*_VIM-5_ and *bla*_VIM-2_). Note that *bla*_VIM-12_ was previously described as a recombinant variant (16); however, the recombinational origin of *bla*_VIM-25_ had not been previously described.

10.1128/mBio.02109-17.1 TEXT S1  GenBank accession numbers. Download TEXT S1, DOCX file, 0.02 MB. Copyright © 2018 Martínez-García et al. 2018 Martínez-García et al. This content is distributed under the terms of the Creative Commons Attribution 4.0 International license.

**FIG 2:**
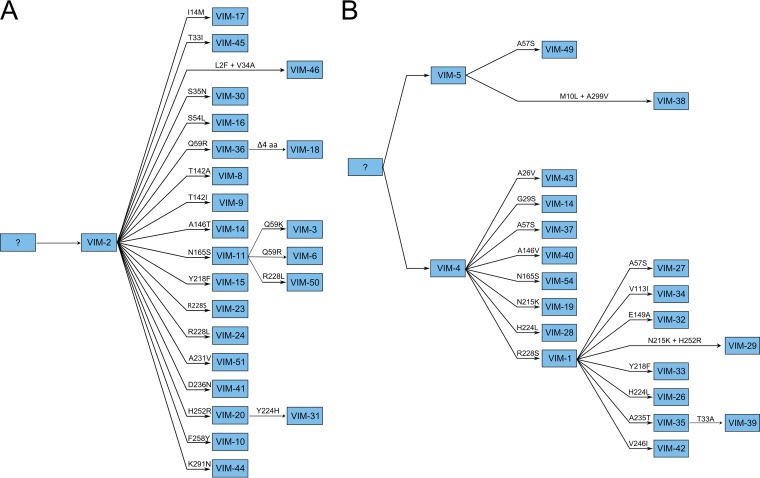
Phylogenetic ancestral reconstruction of two main VIM clusters. (A) VIM-2 cluster. (B) VIM-4/VIM-1 cluster. Using the Mesquite program, version 2.75 (parsimony methods), the ancestral states were inferred. The amino acid changes acquired with respect to the sequence of the ancestor are indicated in the branches.

The reconstruction made using the Network software (Fig. 1B) revealed that the diversifications of *bla*_VIM-4_, *bla*_VIM-1_, and *bla*_VIM-2_ nodes were mainly the results of single, nonsynonymous mutations (52 nonsynonymous changes/63 total changes). In the case of the *bla*_VIM-2_ phylogroup, 20/27 new variants derived directly from *bla*_VIM-2_, carrying only 1 mutation that corresponded to a nonsynonymous change, while in the case of *bla*_VIM-4_/*bla*_VIM-1_, 2 events of diversification could be inferred. From *bla*_VIM-4_, 9 new variants (including *bla*_VIM-1_) harbored only 1 nonsynonymous mutation (9 nonsynonymous changes/9 total changes), whereas this phenomenon was observed in 7/7 variants directly derived from *bla*_VIM-1_. These results suggest a recent and accelerated process of diversification, even though the *bla*_VIM_ ancestors originated a long time ago. BEAST phylogenetic analysis yielded new evidence that the diversification of both *bla*_VIM-2_ and *bla*_VIM-4_ took place around 1987 and 1989, respectively. A second diversification event from a *bla*_VIM-4_ variant, *bla*_VIM-1_, occurred around 1994 (see Fig. S1 in the supplemental material). The evolutionary directionality from *bla*_VIM-4_ to *bla*_VIM-1_ was supported by the parsimony approach and confirmed (see below) by the analyses of experimental evolution under ceftazidime challenge. Phylogenetic reconstructions of ancestral states allowed us to suggest a possible evolutionary scenario for the diversification in *bla*_VIM_ (Fig. 2).

10.1128/mBio.02109-17.5 FIG S1  Phylogenetic analysis obtained using the BEAST evolutionary program, version 1.8. (A) Time-measured phylogeny of *bla*_VIM_ sequences, excluding those with gaps and the recombinant ones. Evolutionary parameters were estimated using a Bayesian Markov chain Monte Carlo (MCMC) method implemented in BEAST. The date used for each sequence in the analysis was the number of years since the earliest sequence isolation. Analysis was performed using the GTR+G model of nucleotide substitution with relaxed lognormal distribution of rates among molecular clock model branches. Analysis was performed with a Bayesian skyline piecewise-constant coalescent tree prior model, using a random starting tree. Two separate MCMC chains were run for 200,000,000 generations with sampling every 20,000 generations and combined after a 10% burn-in. BEAST output was analyzed using Tracer, version 1.5, and the consensus tree was generated using TreeAnnotator. Values greater than 200 for the effective sample size (ESS) were accepted for convergence. *, Posterior probability is >0.9. (B) Bayesian skyline plot (BSP), which calculates the effective breeding population size through time. The BSP is used when the demographic history is not the primary object of interest. BSP was constructed using Tracer, version 1.5, and a stepwise model. The median values of the sampled trace across the chain are represented. Download FIG S1, TIF file, 0.8 MB. Copyright © 2018 Martínez-García et al. 2018 Martínez-García et al. This content is distributed under the terms of the Creative Commons Attribution 4.0 International license.

### Mutations under positive selection, mutagenesis, and susceptibility testing.

The positions under positive selection (ω = *dN*/*dS* > 1) were determined using the BEAST evolutionary program, version 1.8, to reconstruct counts of synonymous (*dS*) and nonsynonymous (*dN*) changes using robust counting (17, 18). In this work, we consider that a mutation has “high evidence” that it will be selected if it has a 99% cumulative posterior density (CPD) interval, “moderate evidence” when it has a 95% CPD interval, and “low evidence” when it has a 90% CPD interval. In all cases, the interval should exclude ω values of 1 (Table S1). The highest ω values will correspond to the strongest evidence of positive selection, and that is indeed the most frequent case, but note that if the CPD interval contains the value ω = 1, positive selection should be discarded.

10.1128/mBio.02109-17.6 TABLE S1  Description of the positions predicted to be under selection pressure using the BEAST evolutionary program, version 1.8. BEAST was used to predict the positions under positive selection (ω > 1) using robust counting. The amino acid changes introduced by the site-directed mutagenesis and the *bla*_VIM_ variants constructed are marked in boldface. ^a^ Positions are classified as high, moderate, and low evidence according to the cumulative posterior density (CPD) intervals, corresponding to 99%, 95% and 90% intervals, respectively. ^b^ Number of VIM refers to the number of VIM enzymes with the corresponding mutation. Download TABLE S1, DOCX file, 0.02 MB. Copyright © 2018 Martínez-García et al. 2018 Martínez-García et al. This content is distributed under the terms of the Creative Commons Attribution 4.0 International license.

Once mutations under positive selection were identified, all these mutations and combinations of pairs of them were constructed by site-directed mutagenesis in *bla*_VIM-2_ and *bla*_VIM-4_/*bla*_VIM-1_ clusters (Table S2). A total of 44 *bla*_VIM_ genes were constructed, 21 from *bla*_VIM-2_ and 23 from *bla*_VIM-4_/*bla*_VIM-1_. Of these, 18 have been previously described in nature (9 from each group).

10.1128/mBio.02109-17.7 TABLE S2  VIM variants constructed by site-directed mutagenesis. (A) VIM-2 cluster. (B) VIM-4 cluster. ^a^ Constructed VIM variants divided into single, double, and triple variants. Those VIM variants described in nature are named with the original name from GenBank. ^b^ Amino acid changes corresponding to nonsynonymous mutations introduced by site-directed mutagenesis are divided into three categories (high, moderate, and low evidence) based on the prediction made using the BEAST evolutionary program, version 1.8. The symbol “X” indicates presence. Download TABLE S2, DOCX file, 0.03 MB. Copyright © 2018 Martínez-García et al. 2018 Martínez-García et al. This content is distributed under the terms of the Creative Commons Attribution 4.0 International license.

Concerning triple mutants, we constructed the only one that has been previously described in natural strains, VIM-29 (derived from VIM-4), associating the mutations R228S, H252R, and N215K. Taking these changes into consideration, we also constructed triple mutants with a similar array of mutations in VIM-2, as an attempt to observe whether similar combinations (R228S, H252R, and Y218F and R228L, H252R, and Y218F) could eventually provide an adaptive advantage, as happened with VIM-29. The MIC values for each VIM variant of the VIM-2 cluster and the VIM-4 cluster are shown in Tables 1 and 2, respectively.

**TABLE 1:** MICs for each variant included in the VIM-2 cluster

Change introduced	Variant	MIC (µg/ml) of^a^:								
		AMP	AMC	PTZ	CTX	CAZ	CEP	ERT	IMI	MER
None	VIM-2	>256	>256	48	16	12	0.5	0.75	1.5	0.75
Q59R	VIM-36	>256	>256	24	12	64	0.125	0.19	2	1.5
Y224H	VIM2_Y224H_	>256	>256	48	24	12	0.25	0.19	1.5	0.38
R228S	VIM-23	>256	>256	32	16	96	1.5	0.25	2	0.75
R228L	VIM-24	>256	>256	24	8	128	1.5	0.047	1.5	1.5
N165S	VIM-11	>256	>256	16	24	128	3	0.25	1	0.75
H252R	VIM-20	>256	>256	8	6	24	0.25	0.094	0.75	0.25
Y218F	VIM-15	>256	>256	64	12	12	0.75	0.094	2	1
Q59R+R228S	VIM-2_Q59R+R228S_	32	64	4	0.25	4	0.064	0.008	0.5	0.125
Q59R+R228L	VIM-2_Q59R+R228L_	>256	>256	12	1.5	>256	0.5	0.25	1.5	0.38
Q59R+N165S	VIM-6	>256	>256	24	2	>256	0.5	0.19	2	1.5
Q59R+H252R	VIM-2_Q59R+H252R_	>256	>256	>256	16	>256	0.19	0.38	8	3
Q59R+Y218F	VIM-2_Q59R+Y218F_	>256	>256	48	4	32	0.25	1.5	6	3
Y224H+H252R	VIM-31	>256	>256	64	16	24	1.5	1.5	4	0.75
R228S+H252R	VIM-2_R228S+H252R_	32	64	4	1.5	2	0.094	0.008	2	1.5
R228S+Y218F	VIM-2_R228S+Y218F_	32	>256	1.5	4	3	0.25	0.016	0.75	0.38
R228L+H252R	VIM-2_R228L+H252R_	>256	>256	12	16	>256	1	0.5	2	1.5
R228L+Y218F	VIM-2_R228L+Y218F_	>256	>256	12	12	24	8	0.5	3	0.75
R228L+N165S	VIM-50	>256	>256	12	12	>256	2	0.047	1.5	1.5
H252R+Y218F	VIM-2_H252R+Y218F_	>256	>256	16	24	24	1	0.75	4	0.75
R228S+H252R+Y218F	VIM-2_R228S+H252R+Y218F_	3	4	2	0.25	0.38	0.047	0.004	0.02	0.02
R228L+H252R+Y218F	VIM-2_R228L+H252R+Y218F_	4	4	2	0.4	0.4	0.094	0.007	0.19	0.032

a The MICs are the medians of the 3 or more values obtained or a MIC value obtained more than once. AMP, ampicillin; AMC, amoxicillin-clavulanate; PTZ, piperacillin-tazobactam; CTX, cefotaxime; CAZ, ceftazidime; CEP, cefepime; ERT, ertapenem; IMI, imipenem; MER, meropenem.

**TABLE 2:** MICs for each variant included in the VIM-4 cluster

Change introduced	Variant	MIC (µg/ml) of^a^:								
		AMP	AMC	PTZ	CTX	CAZ	CEP	ERT	IMI	MER
None	VIM-4	>256	>256	>256	32	16	0.75	0.75	4	0.5
A57S	VIM-37	>256	>256	>256	32	16	0.75	0.19	0.5	0.19
H224L	VIM-28	>256	>256	>256	64	96	1.5	0.5	2	3
R228S	VIM-1	>256	>256	48	24	>256	6	0.125	1	0.25
N165S	VIM-54	>256	>256	>256	64	>256	8	0.75	3	2
N215K	VIM-19	>256	>256	>256	32	32	1.5	1.5	4	0.5
H252R	VIM-4_H252R_	>256	>256	>256	48	64	2.5	0.5	2	0.75
Y218F	VIM-4_Y218F_	>256	>256	>256	>256	24	0.75	0.75	4	1.5
A57S+H224L	VIM-4_A57S+H224L_	>256	>256	8	4	0.5	0.032	0.5	0.75	0.38
A57S+R228S	VIM-27	>256	>256	>256	>256	>256	12	1	8	8
A57S+N215K	VIM-4_A57S+N215K_	>256	>256	>256	24	64	2	1.5	2	6
A57S+H252R	VIM-4_A57S+H252R_	>256	>256	>256	6	1.5	0.064	0.064	2	0.5
A57S+Y218F	VIM-4_A57S+Y218F_	>256	>256	4	4	0.5	0.032	0.064	0.38	0.125
H224L+R228S	VIM-26	>256	>256	>256	32	>256	4	0.094	1.5	0.19
H224L+N215K	VIM-4_H224L+N215K_	>256	>256	>256	>256	>256	1	0.75	1.5	1
H224L+H252R	VIM-4_H224L+H252R_	>256	>256	>256	>256	>256	2	3	3	2
H224L+Y218F	VIM-4_H224L+Y218F_	12	12	3	2	0.25	0.023	0.006	0.19	0.032
R228S+N215K	VIM-4_R228S+N215K_	>256	>256	>256	>256	>256	24	0.25	1.5	0.75
R228S+H252R	VIM-4_R228S+H252R_	>256	>256	>256	>256	>256	32	0.38	3	1
R228S+Y218F	VIM-33	>256	>256	>256	>256	>256	32	0.5	3	3
N215K+H252R	VIM-4_N215K+H252R_	>256	>256	>256	64	24	2	0.38	2	0.75
N215K+Y218F	VIM-4_N215K+Y218F_	>256	>256	>256	>256	32	1.5	1	4	3
H252R+Y218F	VIM-4_H252R+Y218F_	24	64	4	2	0.5	0.023	0.016	0.38	0.047
R228S+N215K+H252R	VIM-29	>256	>256	>256	64	>256	12	1	2	2

a The MICs are the medians of the 3 or more values obtained or a MIC value obtained more than once. AMP, ampicillin; AMC, amoxicillin-clavulanate; PTZ, piperacillin-tazobactam; CTX, cefotaxime; CAZ, ceftazidime; CEP, cefepime; ERT, ertapenem; IMI, imipenem; MER, meropenem.

### VIM-2 phylogroup.

**FIG 3:**
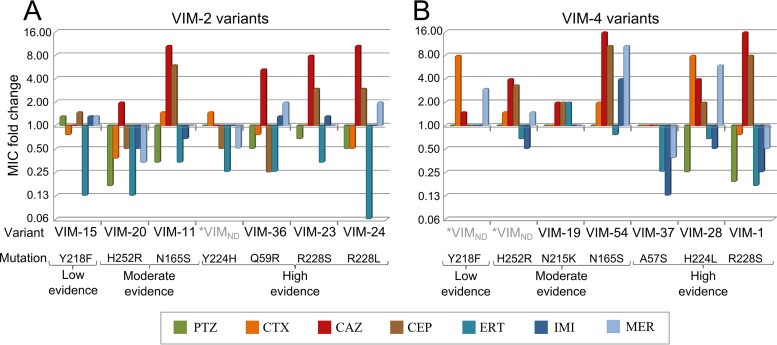
Representation of the fold changes in MICs for constructed single variants with respect to the MICs for the original VIM enzymes. (A) MICs for single mutants derived from VIM-2. (B) MICs for single mutants derived from VIM-4. According to the mutation introduced in each case and following the nomenclature used in Table S1 in the supplemental material, the variants constructed are identified as having high, moderate, and low evidence for being under positive selection according to the cumulative posterior density (CPD) intervals, corresponding to 99%, 95%, and 90% intervals, respectively. The VIM variants previously described in nature are shown with the GenBank numbering. Asterisks indicate VIM variants that have not been described (ND). PTZ, piperacillin-tazobactam; CTX, cefotaxime; CAZ, ceftazidime; CEP, cefepime; ERT, ertapenem; IMI, imipenem; MER, meropenem.

In the first round of site-directed mutagenesis, when single mutations were introduced in *bla*_VIM-2_, only the MICs of ceftazidime showed important increases in almost all mutants. In addition, those mutants carrying the mutations predicted with high evidence to be under positive selection yielded the highest increases in the MICs of ceftazidime. It is also remarkable that the carbapenem MIC values hardly increased or even decreased, especially for ertapenem. In fact, an antagonist effect was found between the MICs for ceftazidime and ertapenem or piperacillin-tazobactam (Fig. 3A). This pleiotropic antagonism was more drastic when the R228L change was present, corresponding to the VIM-24 variant, which yielded the highest MIC value for ceftazidime with the lowest MIC value for ertapenem. Two variants, VIM-2_Y218F_ and VIM-2_Y224H_, did not show evidence of pleiotropic antagonism, and neither contributed to increase the MIC. All variants have been described in nature, except VIM-2_Y224H_.

In the second round of site-directed mutagenesis in the *bla*_VIM-2_ variants, the constructs with the R228S and R228L double changes exhibited very different results. All mutants carrying R228S yielded a negative effect on the MICs against all β-lactam antibiotics tested. On the other hand, the four double mutants carrying R228L had increased MICs against ceftazidime (except for the combination of R228L and Y218F). The pleiotropic antagonism was partially reverted, because the MICs of ertapenem increased until reaching the original value for wild-type VIM-2, but the same did not occur with the MICs of piperacillin-tazobactam, which continued to decrease. In nature, only one double mutant carrying R228L has been described, corresponding to VIM-50 (VIM-2 with the R228L and N165S changes). This variant could be selected from VIM-24 (VIM-2_R228L_) or VIM-11 (VIM-2_N165S_), but in both cases, the MICs of ceftazidime showed the highest increases once again (Table 1). Among the remaining double mutants derived from VIM-2 that were detected in nature, VIM-6 yielded a reduction in the MIC of cefotaxime compared with those of VIM-36 (VIM-2_Q59R_) and VIM-11. If the ancestor of this double variant was VIM-36, a 4-fold increase in the MIC of ceftazidime could be observed, but if the ancestral mutant was VIM-11, ≥2-fold increases in the MICs of ceftazidime and carbapenems could explain the selection. Another double mutant detected in nature was VIM-31, carrying the H252R and Y224H changes. The introduction of Y224H in VIM-20 (VIM-2_H252R_) increased the MICs of all β-lactam antibiotics tested except ceftazidime, reverting the pleiotropic antagonism observed for piperacillin-tazobactam and ertapenem. Moreover, VIM-31 yielded a higher MIC for imipenem than the other double mutants described. However, the highest MICs for imipenem were observed in variants carrying the combinations of H252R plus Q59R and Q59R plus Y218F. These variants have not been described in nature, although single mutants carrying each of these changes (VIM-20, VIM-36, and VIM-15) have been described, suggesting that the carbapenems have not greatly affected the selection and diversification of VIM enzymes.

Although triple mutants derived from VIM-2 have not yet been described, two triple mutants derived from VIM-2 were constructed in this work by site-directed mutagenesis, carrying a combination of mutations similar to those of VIM-29 (the unique triple VIM mutant described in nature). These mutants were VIM-2 with R228S/L, H252R, and Y218F changes (whereas VIM-29 has the R228S, H252R, and N215K changes). Interestingly, in both cases, the MICs against all β-lactam antibiotics tested were consistently reduced.

### VIM-4/VIM-1 phylogroup.

A similar approach was used for measuring the effects of single, double, and triple mutations in *bla*_VIM-4_ (Table S2). Seven variants carrying a single change with respect to VIM-4 were analyzed, five of them corresponding to variants described in nature. Similarly to the VIM-2 reconstruction, one of the antibiotics whose MICs were most affected was ceftazidime, especially in the variants carrying the R228S and H224L mutations, corresponding to VIM-1 and VIM-28, respectively. Moreover, the pleiotropic antagonism between ceftazidime and ertapenem or piperacillin-tazobactam was also observed. In the case of the N165S mutation (VIM-54), the increased MIC for ceftazidime was similar to that for VIM-1, but the pleiotropic antagonism was not observed (Fig. 3B). In contrast, the Y218F mutation, which was without effect in the VIM-2 background, showed increased MICs for cephalosporins (especially cefotaxime), while the N215K mutation, which was not tested in the VIM-2 background and had moderate evidence of selection, yielded discrete increases in the MICs of ertapenem and ceftazidime.

In the case of double mutants constructed from previously described VIM-4 variants, special attention was paid to the variants derived from those single mutants that yielded major changes in the MICs for the β-lactams tested (VIM-1 and VIM-28). Five new mutants carrying the R228S change (VIM-1) were constructed, three of which were previously described in nature. All combinations reverted the pleiotropic antagonism observed for piperacillin-tazobactam until reaching the MIC of VIM-4 wild type, without a decrease in the hydrolytic activity for ceftazidime. Moreover, all combinations showed positive epistasis against cefotaxime, except the double mutant VIM-26, carrying two changes with high evidence of being under positive selection (R228S and H224L). However, for carbapenems, only VIM-26 among the constructed variants showed no hydrolytic advantages for these compounds. Therefore, according to the pattern of MICs obtained for the three natural variants (VIM-26, VIM-27, and VIM-33) and common to the variants that were not described, the diversification derived from VIM-1 (Fig. 2B) could be more related to reverting the hydrolytic activity against piperacillin-tazobactam and eliminating the pleiotropic antagonism than to effects with regard to oxyimino-cephalosporins or carbapenems. On the other hand, only the H252R mutation introduced in VIM-28 (corresponding to VIM-4_H224L_) yielded an increase in the MICs for practically all β-lactams, especially cefotaxime, ceftazidime, and ertapenem. The other double mutants yielded negative effects, suggesting that these combinations could hardly be selected in nature. A diversification event from VIM-28 was not observed, but we suspect that a double mutant carrying H224L and H252R could be selected in the future.

We also constructed a triple mutant, VIM-29, which had three changes with respect to VIM-4 (R228S, N215K, and H252R). There are several trajectories to reach VIM-29, from VIM-1 or from VIM-19. We would like to underline that the single H252R mutation has not been described in nature, whereas R228S and N215K correspond to VIM-1 and VIM-19, respectively, suggesting that the initial diversification events could be more related to cephalosporins than to carbapenems.

### Experimental evolution.

At first sight, the evolution of VIM β-lactamases toward more-efficient enzymes should be driven by carbapenems. However, according to the results described above, the role of oxyimino-cephalosporins had to be explored. This was the goal of our experimental-evolution assays (see Materials and Methods). In the results reported below, imipenem was used as a representative drug for carbapenems. However, experiments were also performed with meropenem and ertapenem as selective agents. These results are not presented, as the mutants obtained do not differ from those obtained from imipenem selection.

In the case of VIM-2, when the selector antibiotic was a carbapenem (imipenem) or an extended-spectrum cephalosporin (cefotaxime), we never obtained VIM-2 variants with higher hydrolytic activities than the wild type. In contrast, when ceftazidime was used as the selector antibiotic in different approaches, VIM-11 (corresponding to VIM-2_N165S_) was always selected. Moreover, other mutants were also selected. Two of the four positions predicted with high evidence to be under positive selection were selected in the evolution experiments with ceftazidime. These mutations were Q59R (corresponding to VIM-36) and R228L (corresponding to VIM-24).

In the experimental-evolution assays of VIM-4, results similar to those from the VIM-2 serial passages were obtained. No mutants were detected in cultures containing imipenem or cefotaxime; in contrast, several types of mutants were selected in the different serial-passage experiments with ceftazidime (Table S3). The R228S mutation, corresponding to VIM-1, was obtained at increasing concentrations of ceftazidime. We also obtained the N165S mutation, identified as VIM-54, and a not-described mutant carrying the Q59R change. A double mutant carrying N165S and Q59R was detected at increasing concentrations of ceftazidime and a fixed concentration of imipenem. This variant has not yet been described in nature but corresponds to *bla*_VIM-6_ in the VIM-2 cluster. No mutants were obtained in serial-passage experiments when *bla*_VIM-1_ was used as the wild type.

10.1128/mBio.02109-17.8 TABLE S3  Mutations obtained in the different serial-passage experiments. The mutations found are classified as having high evidence (99% cumulative posterior density interval that excludes ω = 1), moderate evidence (95% cumulative posterior density interval that excludes ω = 1), and low evidence (90% cumulative posterior density interval that excludes ω = 1) that they will be under selection according to the Bayesian prediction made using the BEAST evolutionary program, version 1.8. The percentage indicated for each mutation refers to the number of lineages that had the change with respect to the total number of lineages involved in the experiment. ^a^ At increasing concentrations of CAZ and a fixed concentration of IMI, the mutation Q59R in VIM-4 was selected in combination with N165S (nondescribed double mutant). CAZ, ceftazidime; IMI, imipenem; MER, meropenem; ERT, ertapenem; CTX, cefotaxime. Download TABLE S3, DOCX file, 0.03 MB. Copyright © 2018 Martínez-García et al. 2018 Martínez-García et al. This content is distributed under the terms of the Creative Commons Attribution 4.0 International license.

## DISCUSSION

The phylogenetic reconstructions using all available *bla*_VIM_ nucleotide sequences in GenBank indicate that the current VIM family is structured in four phylogroups (Fig. 1A) and that the gene variations detected resulted from three large, explosive events of diversification that occurred recently (Fig. 2; see Fig. S1 in the supplemental material). In this work, three different strategies were applied to understand the selective forces triggering the recent diversification of VIM β-lactamases. First, bioinformatics analysis predicted several positions to be under positive selection, suggesting an accelerated process of diversification. Several changes, classified according to the evidence levels of positions under positive selection, were analyzed. Positions 59, 224, and 228 (classified as having high levels of evidence for selection) were identified, confirming previous observations (19, 20); however, we were also able to detect a high level of evidence for position 57. We also selected other mutations with only moderate evidence of selection (positions 165, 215, and 252) but with possible adaptive significance, as they were found in more than one natural VIM variant and in different evolutionary trajectories.

A total of 44 mutants were constructed by site-directed mutagenesis, including those mutations previously identified in VIM-2 and VIM-4 backgrounds. Surprisingly, the oxyimino-cephalosporins, especially ceftazidime, but not the carbapenems were the antibiotics whose MICs were most affected by the introduced changes (Fig. 3). For instance, among the single mutants derived from VIM-2, all those previously described in nature (VIM-20, VIM-36, VIM-23, VIM-24, and VIM-11) increased the ceftazidime MICs without any effect on carbapenems. Similar results were obtained in the case of VIM-4. In both models, position 228 yielded the most-dramatic effect on the increase in MICs. This position is in the neighborhood of the active site, and several authors have suggested the important role of this position for the hydrolytic activity in VIM β-lactamases (19–21), possibly to accommodate bigger or hydrophobic substrates (22). Recently, and in agreement with our own results, site-specific mutagenesis in VIM-2 revealed 4- to 6-fold increases in the MICs of ceftazidime and cefepime (23). Similar observations have been obtained for VIM-4, where the substitution in position 228 produced 16-fold increases in the MICs of ceftazidime and cefepime, with reductions in the MICs of carbapenems (15), again a pattern comparable to that obtained in the present work. Position 224 is also close to the active site (20), and the presence of a tyrosine in VIM-2 is important to improve the efficiency of hydrolysis of ceftazidime and cefepime but is less effective toward carbapenems (24). Consistent with this, we did not observe any changes in MICs with the introduction of the Y224H change in VIM-2. In contrast, in the case of VIM-4, the H224L change increased the MICs to oxyimino-cephalosporins but had a negative effect for carbapenems, except meropenem. Mutational changes in other positions remote from the enzyme active site have also been detected in natural strains, such as N165S (25), N215K (22), or H252R (26). These mutations were identified as having moderate evidence of being under positive selection. The variants carrying the N165S change (VIM-11) displayed better catalytic efficiencies for oxyimino-cephalosporins, especially for ceftazidime, with 4-fold MIC increases compared with the MICs of VIM-2. Meanwhile, the H252R change was previously associated with increased activity against carbapenems (26), as suggested by the MICs in our work (VIM-29). In general, those positions suggested in the bioinformatics analysis to be under positive selection, and which have been selected in nature, showed pleiotropic antagonism not only between ceftazidime and carbapenems (especially ertapenem) but also between ceftazidime and piperacillin-tazobactam (Fig. 3). However, double mutants derived from these variants carrying those positions with high evidence of being under positive selection partially or completely reverted the pleiotropic antagonism. In the case of VIM-2 variants, this effect was more evident in the case of carbapenem activity, but in VIM-4 variants, only the original MICs for piperacillin-tazobactam were completely reverted in those combinations carrying the R228S change.

Note that we use above the term “catalytic efficiency” as the main driver of the resistance phenotype, but it is to be noticed that MIC levels might not only reflect the catalytic efficiency of the mutants. Reduced MICs might correlate with lower stability of the enzyme, but probably enzyme efficiency is far more important in the evolution of resistance (27, 28). Reduced protein levels resulting from lower gene expression might also influence the selectable resistance phenotype (13). In fact, in our serial-passage experiments, we detected an E30A change in VIM-4. This previously uncharacterized mutation could influence the excretion of the enzyme to the periplasmic space (29), but we have not studied this evolutionary possibility in detail, although the occurrence of several variants carrying mutations between positions 29 and 35 reveals the important role of this region in VIM evolution. Our work is focused on amino acid changes exclusively in the VIM protein and how these changes, presumably leading to changes in enzymatic effectiveness (mostly catalytic efficiency), have been selected by antibiotic exposure.

The experimental-evolution approach, based on challenging cultures of a strong Escherichia coli mutator strain carrying VIM-2 and VIM-4 with different β-lactam antibiotics, yielded evolved variants only when the selector agent was ceftazidime and never in cultures containing cefotaxime or any of the carbapenems (imipenem, meropenem, or ertapenem). This result could suggest that ceftazidime constitutes the antibiotic with the strongest influence in the diversification of VIM β-lactamases. For instance, the VIM-11 variant (VIM-2_N165S_), which was obtained under ceftazidime selection, had increased ceftazidime (10-fold) and cefepime (6-fold) MICs but reduced hydrolytic activity against carbapenems (3-fold) and piperacillin-tazobactam (6-fold) (pleiotropic antagonism). An identical mutation was detected with ceftazidime when VIM-4 was used as the original enzyme (corresponding to VIM-54). This variant yielded increased MICs of ceftazidime (16-fold) and cefepime (10-fold), but pleiotropic antagonism was not detectable with respect to the original VIM-4. It might be expected that the mutation Y218F could be selected in the experimental evolution with cefotaxime. However, it was not obtained or detected, probably because of the bottlenecks generated in the experimental-evolution assays (both in serial passages and the numbers of clones sequenced). In a second scenario, using a fixed concentration of imipenem and increasing concentrations of ceftazidime, a double mutant (N165S and Q59R) was selected in the VIM-4 evolution. This variant has not been previously described as derived from VIM-4, but an identical combination of mutations has been described in VIM-2 in nature (corresponds to VIM-6). This VIM-2_N165S+Q59R_ mutant showed an increased MIC for ceftazidime but maintained similar MICs for carbapenems and was negatively affected in its susceptibility to cefotaxime. This pattern was also observed in other double mutants, such as those with Q59R plus Y218F or Q59R plus H252R changes. However, none of these other variants have been detected in nature, suggesting a discrete effect only of carbapenems in the diversification and selection of new VIM variants. The scarce available information about site-directed mutagenesis in VIM β-lactamases, restricted to a few mutants, is consistent with our observations suggesting the impact of cephalosporins, especially of ceftazidime, in the selection of new variants (15, 30). Our extensive study, comparatively analyzing different VIM mutants in an isogenic context, reveals that ceftazidime could be more responsible than any of the carbapenems tested for the evolution and diversification of carbapenemase enzymes.

The effect of oxyimino-cephalosporins in the diversification of other types of β-lactamases, such as TEM (13) and CTX-M (12), has been extensively studied. A close examination of these cases, in comparison with the results presented in this work, suggests that ceftazidime seems to be a key player in the selection of new variants in different groups of β-lactamases. For instance, ESBL-type CTX-M enzymes and the ancestral CTX-M variants, such as CTX-M-3, are able to hydrolyze cefotaxime but not ceftazidime, and they evolved toward variants with the capacity to hydrolyze cefotaxime and ceftazidime efficiently (12). A similar result was also observed in serine carbapenemases, such as KPC, where the evolved variants yielded more highly increased catalytic efficiencies for ceftazidime than for the carbapenems (14).

Why might ceftazidime have promoted, more efficiently than other β-lactam antibiotics, the selection of novel variants in both narrow-spectrum and extended-spectrum β-lactamases or serine and metallo-carbapenemases? First to be noticed is that the selective advantage of the new variants depends on the difference in MICs provided by the original enzyme and the novel variant. Those β-lactams less efficiently hydrolyzed by a β-lactamase type should be the best candidates to contribute to the diversification. It could help us explain why cefotaxime and ceftazidime could have contributed to the diversification in TEM and CTX-M, but it is less clear in the case of carbapenemases, such as KPC and VIM. Second, as antibiotic drug consumption is a major driver of antibiotic resistance, the novel epidemiological landscapes might also contribute to explaining the explosive diversification of carbapenemases, such as that of VIM types. In the largest study about antibiotic consumption, consumption rates for the carbapenems were increased by 45% and for cephalosporins by 93%; moreover, the largest absolute increase in consumption was observed in this group (3). In our view, both drugs were frequently used over recent decades in the same areas, such as hospitals. The increased consumption of carbapenems, related to the global spread of ESBLs, has contributed to the selection of bacteria containing β-lactamases with the capacity to hydrolyze carbapenems with different efficiencies. In this context, the simultaneous or sequential exposure to ceftazidime in the same ward or environment could have facilitated the secondary diversification and further spread of carbapenemase variants with higher efficiencies against extended-spectrum β-lactams. The initial premise would suggest that it was mostly the carbapenems that might have contributed to the development of more-efficient new variants; however, the impact of these β-lactam antibiotics was very weak in this process of diversification, although the hydrolytic capacity was low. Third, ceftazidime might also contribute to the mutational yield of target organisms (due to efficient SOS and DNA polymerase IV error-prone polymerase induction) (31, 32) to a much higher degree than carbapenems (33, 34). The data shown in this work, together with previous results, reveal the main role of ceftazidime not only in the diversification of VIM enzymes but also of all β-lactamases studied, increasing the spectrum of hydrolytic activities of the new variants.

## MATERIALS AND METHODS

### Phylogenetic analysis.

In the first stage, all available *bla*_VIM_ nucleotide sequences (801 nucleotides) were obtained from GenBank using BLAST. Of the 503 sequences downloaded, 61 showed some difference in their nucleotide compositions, corresponding to 49 VIM types (VIM-1 to VIM-54). Nucleotide sequences were translated and aligned using the ClustalW algorithm implemented in MEGA, followed by manual editing to discard sequencing and alignment errors. Maximum-likelihood (ML) trees were constructed using PhyML 3.0 (35), with the nucleotide substitution model (GTR+G) inferred by using jModelTest (36). The bootstrap test (1,000 replicates) was used to calculate branch support. Only support values of >90% were considered statistically significant. To determine possible recombination events, sequences were analyzed using Recombination Detection Program 3beta27 (RDP3beta27) (37). New phylogenetic analyses were performed according to the proposed breakpoint position(s) in those suspected recombinant sequences to confirm the putative recombination events detected. Mesquite version 2.75 was used to infer the ancestral states using parsimony methods (http://mesquiteproject.org).

Mutations involved in adaptive processes under positive selection were identified according to the rule ω = (*dN*/*dS*) > 1, where *dN* corresponds to nonsynonymous changes (leading to a new amino acid in the protein) and *dS* to synonymous changes (maintaining the original amino acid). ω values of <1 indicate purifying selection and ω values of ~1 represent neutral evolution, whereas ω values of >1 imply positive selection. The estimated evidence of positive selection for each position in the VIM sequences was inferred using the BEAST (Bayesian Evolutionary Analysis by Sampling Trees) software package, version 1.8. Inferences are obtained about rooted, time-measured phylogenies without conditioning on a single tree topology, implementing a family of Markov chain Monte Carlo (MCMC) algorithms to average over tree space, so that each tree is weighted in proportion to its posterior probability (38). Using this analysis, it is possible to infer for a given mutation the probability that it will come under positive selection based on ω estimation and uncertainty calculation. In short, the evolutionary significance of each codon (ω) is estimated within a cumulative posterior density (CPD) interval, an interval in the domain of a posterior probability distribution, or a predictive distribution (18). Network 5.0.0.1 (Fluxux Technology) was used to reconstruct phylogenetic networks. In both cases, all sequences except those with gaps and the recombinant ones were used. The accession numbers of these sequences are shown in Text S1 in the supplemental material. The Network software was developed to reconstruct all possible shortest and least-complex phylogenetic trees (all maximum-parsimony [MP] trees) from a given data set. The median-joining (MJ) algorithm identifies groups of closely related types and introduces hypothesized ancestral types, in order to unite the types into a parsimonious network (39).

### Experimental reconstruction.

The first step was the amplification of *bla*_VIM-2_ and *bla*_VIM-4_ (see Text S2 for descriptions of primers). The PCR amplicons obtained were cloned in pCR-Blunt II-TOPO (Km^r^) and transformed into Escherichia coli TOP10 using the ZeroBlunt TOPO PCR cloning kit (Invitrogen, Cergy-Pontoise, France). Plates containing 40 µg/ml of ampicillin, 50 µg/ml of kanamycin, and 80 µg/ml of X-Gal (5-bromo-4-chloro-3-indolyl-β-d-galactopyranoside)–IPTG (isopropyl-β-d-thiogalactopyranoside) were used for the selection of E. coli strains carrying the *bla*_VIM_ genes.

10.1128/mBio.02109-17.2 TEXT S2  Cloning and transformation. Download TEXT S2, DOCX file, 0.02 MB. Copyright © 2018 Martínez-García et al. 2018 Martínez-García et al. This content is distributed under the terms of the Creative Commons Attribution 4.0 International license.

Single, double, and triple *bla*_VIM_ variants were constructed from *bla*_VIM-2_ and *bla*_VIM-4_ by site-directed mutagenesis according to three levels of evidence of the positions being under positive selection as inferred by using BEAST, version 1.8 (Table S1). In the case of *bla*_VIM-4_, the changes introduced were as follows: (i) A57S (Ala57Ser), H224L (His224Leu), and R228S (Arg228Ser) with high evidence, (ii) N165S (Asn165Ser), N215K (Asn215Lys), and H252R (His252Arg) with moderate evidence, and (iii) Y218F (Tyr218Phe) with low evidence. Although the Y218F mutation was predicted to be under positive selection with a 90% CPD interval, it was also included because it was found in the two main clusters. The changes introduced in *bla*_VIM-2_ were identical to those introduced in *bla*_VIM-4_, except for A57S and N215K, which were replaced by Q59R (Gln59Arg) and R228L (Arg228Leu). Moreover, the mutation in position 224 was different (Y224H [Tyr224His]). The construction of the mutants was performed using the NZY mutagenesis kit (NZYTech, Lisbon, Portugal). The presence of the desired mutation was confirmed by sequencing analysis. All the primers used are described in Table S4, and the mutagenesis protocol used is shown in Text S3. In order to get all the *bla*_VIM_ genes in an isogenic context, the plasmids constructed were also transformed into E. coli TOP10.

10.1128/mBio.02109-17.3 TEXT S3  Site-directed-mutagenesis protocol. Download TEXT S3, DOCX file, 0.02 MB. Copyright © 2018 Martínez-García et al. 2018 Martínez-García et al. This content is distributed under the terms of the Creative Commons Attribution 4.0 International license.

10.1128/mBio.02109-17.9 TABLE S4  Primers used for the mutagenesis of *bla*_VIM-2_ and *bla*_VIM-4_. Download TABLE S4, DOCX file, 0.02 MB. Copyright © 2018 Martínez-García et al. 2018 Martínez-García et al. This content is distributed under the terms of the Creative Commons Attribution 4.0 International license.

### Experimental evolution.

The pCR-Blunt II-TOPO plasmids containing the *bla*_VIM-4_, *bla*_VIM-1_, and *bla*_VIM-2_ genes were introduced into E. coli XL1-Red, a hypermutator strain due to the deletion or modification of three genes implicated in the DNA repair pathways (*mutD*, *mutS*, and *mutT*) (Agilent Technologies, Santa Clara, CA).

Eleven independent colonies each of E. coli XL1-Red harboring the *bla*_VIM_ genes were inoculated into 2 ml of Luria broth (Conda Laboratories, Madrid, Spain) containing 50 µg/ml of kanamycin and 250 µg/ml of cloxacillin to inhibit the E. coli AmpC and were submitted to serial passages with different antibiotics and selection conditions, as follows: (i) increasing concentrations (2-fold) of ceftazidime, cefotaxime, or a carbapenem (imipenem, meropenem, or ertapenem); (ii) increasing concentrations of ceftazidime (2-fold) and a fixed, subinhibitory concentration of imipenem; and (iii) increasing concentrations (2-fold) of imipenem and ceftazidime sequentially over alternative days. For a more detailed description of these experimental-evolution trials, see Text S4. Analysis of mutants was performed at the highest antibiotic concentration at which bacterial growth was detected.

10.1128/mBio.02109-17.4 TEXT S4  Experimental-evolution details. Download TEXT S4, DOCX file, 0.6 MB. Copyright © 2018 Martínez-García et al. 2018 Martínez-García et al. This content is distributed under the terms of the Creative Commons Attribution 4.0 International license.

### Susceptibility testing.

Susceptibility testing was performed by using MIC strips (Liofilchem, Roseto degli Abruzzi, Italy) on Mueller-Hinton agar plates at 37°C. The MICs were determined at least three times for each E. coli TOP10 strain harboring the constructed plasmids. A control strain corresponding to E. coli TOP10 containing a wild-type plasmid without the *bla*_VIM_ insert was used. Ampicillin (AMP), amoxicillin-clavulanate (AMC), piperacillin-tazobactam (PTZ), cefotaxime (CTX), ceftazidime (CAZ), cefepime (CEP), imipenem (IMI), meropenem (MER), and ertapenem (ERT) were the antimicrobial agents tested.

### Data availability.

The GenBank (https://www.ncbi.nlm.nih.gov/nucleotide) accession numbers and the references of the 57 *bla*_VIM_ sequences used for the phylogenetic analysis are listed in Text S1 (last time accessed July 2017). The programs used to perform the phylogenetic analysis were the following: https://www.megasoftware.net/ (MEGA), http://www.atgc-montpellier.fr/phyml/ (PhyML), https://github.com/ddarriba/jmodeltest2 (jModeltest), http://darwin.uvigo.es/rdp/rdp.html (RDP), http://tree.bio.ed.ac.uk/software/ (BEAST/Tracer/FigTree), http://mesquiteproject.org/ (MESQUITE), and http://www.fluxus-engineering.com/ (Network). Moreover, the data sets generated and/or analyzed during the current study are available from the corresponding author on reasonable request.
