# Supplementary material for: Ceftazidime Is the Key Diversification and Selection Driver of VIM-Type Carbapenemases
Source: mBio. 2018 May 8;9(3):e02109-17. doi: 10.1128/mBio.02109-17 (PMC5941070; doi:10.1128/mBio.02109-17)
Supplement: TEXT S1 [file mbo002183862s1.docx]

**Text S1. GenBank accession numbers.**

| ***bla*VIM** | **Accession number** | **Reference** |
| --- | --- | --- |
| *bla*VIM-1 | KY020154 | Unpublished^*^ |
| *bla*VIM-2 | KX889311 | Unpublished^*^ |
| *bla*VIM-2 | AJ511268 | 1 |
| blaVIM-2 | DQ287356 | 2 |
| blaVIM-2 | AY294333 | 3 |
| *bla*VIM-2like | EF394441 | Unpublished^*^ |
| *bla*VIM-2like | KP754010 | 4 |
| *bla*VIM-2like | KR337991 | 5 |
| *bla*VIM-2like | HQ454494 | Unpublished^*^ |
| *bla*VIM-2like | GU137304 | Unpublished^*^ |
| *bla*VIM-2like | EU571945 | Unpublished^*^ |
| *bla*VIM-3 | NG_050356 | 6 |
| *bla*VIM-4 | LC169580 | Unpublished^*^ |
| *bla*VIM-4like | FM179466 | 7 |
| *bla*VIM-4like | AJ585042 | 8 |
| *bla*VIM-5 | LC169578 | Unpublished^*^ |
| *bla*VIM-6 | NG_050380 | 9 |
| *bla*VIM-8 | NG_050382 | 10 |
| *bla*VIM-9 | NG_050383 | Unpublished^*^ |
| *bla*VIM-10 | NG_050337 | Unpublished^*^ |
| *bla*VIM-11 | NG_050338 | 11 |
| *bla*VIM-13 | NG_050340 | 12 |
| *bla*VIM-14 | EF055455 | Unpublished^*^ |
| *bla*VIM-14 | NG_050341 | Unpublished^*^ |
| *bla*VIM-15 | NG_050342 | 13 |
| *bla*VIM-16 | NG_050343 | 13 |
| *bla*VIM-17 | NG_050344 | 14 |
| *bla*VIM-19 | LC169563 | Unpublished^*^ |
| *bla*VIM-20 | NG_050348 | Unpublished^*^ |
| *bla*VIM-23 | LC169586 | Unpublished^*^ |
| *bla*VIM-24 | NG_050350 | 15 |
| *bla*VIM-26 | LC169582 | Unpublished^*^ |
| *bla*VIM-27 | NG_050353 | 16 |
| *bla*VIM-28 | NG_050354 | Unpublished^*^ |
| *bla*VIM-29 | NG_050355 | Unpublished^*^ |
| *bla*VIM-30 | NG_050357 | Unpublished^*^ |
| *bla*VIM-31 | NG_050358 | 17 |
| *bla*VIM-32 | NG_050359 | Unpublished^*^ |
| *bla*VIM-33 | LC169581 | Unpublished^*^ |
| *bla*VIM-34 | NG_050361 | 18 |
| *bla*VIM-35 | NG_050362 | Unpublished^*^ |
| *bla*VIM-36 | NG_050363 | Unpublished^*^ |
| *bla*VIM-37 | NG_050364 | Unpublished^*^ |
| *bla*VIM-38 | NG_050365 | 19 |
| *bla*VIM-39 | NG_050366 | 20 |
| *bla*VIM-40 | NG_050368 | Unpublished^*^ |
| *bla*VIM-41 | NG_050369 | Unpublished^*^ |
| *bla*VIM-42 | NG_050370 | Unpublished^*^ |
| *bla*VIM-43 | NG_050371 | Unpublished^*^ |
| *bla*VIM-44 | NG_050372 | Unpublished^*^ |
| *bla*VIM-45 | NG_050373 | Unpublished^*^ |
| *bla*VIM-46 | NG_050374 | Unpublished^*^ |
| *bla*VIM-47 | NG_050375 | Unpublished^*^ |
| *bla*VIM-49 | NG_050376 | Unpublished^*^ |
| *bla*VIM-50 | NG_050378 | Unpublished^*^ |
| *bla*VIM-51 | NG_050379 | Unpublished^*^ |
| *bla*VIM-54 | KY508061 | Unpublished^*^ |
| * Unpublised sequence. Available in GenBank (**www.ncbi.nlm.nih.gov/nucleotide**) | | |

**References**

1. Riccio ML, Docquier JD, Dell’Amico E, Luzzaro F, Amicosante G, Rossolini GM. 2003. Novel 3-N-aminoglycoside acetyltranferase gene, aac(3)-Ic, from a *Pseudomonas aeruginosa* integron. Antimicrob Agents Chemother 47:1746-1748.
2. Jeong JH, Shin KS, Lee JW, Park EJ, Son SY. 2009. Analysis of a novel class 1 integron containing metallo-beta-lactamase gene VIM-2 in *Pseudomonas aeruginosa*. J Microbiol 47:753-759.
3. Yatsuyanagi J, Saito S, Harata S, Suzuki N, Ito Y, Amano K, Enomo K. 2004. Class 1 integron containing metallo-beta-lactamase gene *bla*VIM-2 in *Pseudomonas aeruginosa* clinical strains isolated in Japan. Antimicrob Agents Chemother 48:626-628.
4. Viedma E, Estepa V, Juan C, Castillo-Vera J, Rojo-Bezares B, Seral C, Castillo FJ, Saenz Y, Torres C, Chaves F, Oliver A. 2014. Comparison of local features from two Spanish hospitals reveals common and specific traits at multiple levels of the molecular epidemiology of metallo-beta-lactamase-producing *Pseudomonas* spp. Antimicrob Agents Chemother 58:2454-2458.
5. Wright LL, Turton JF, Hopkins KL, Livermore DM, Woodford N. 2015. Genetic environment of metallo-beta-lactamase genes in *Pseudomonas aeruginosa* isolates from the UK. J Antimicrob Chemother 70:103-110.
6. Yan JJ, Hsueh PR, Ko WC, Luh KT, Tsai SH, Wu HM, Wu JJ. 2001. Metallo-beta-lactamase in clinical *Pseudomonas* isolates in Taiwan and identification of VIM-3, a novel variant of the VIM-2 enzyme. Antimicrob Agents Chemother 45:2224-2228.
7. Patzer JA, Walsh TR, Weeks J, Dzierzanowska D, Toleman MA. 2009. Emergence and persistence of integron structures harbouring VIM genes in the Children’s Memorial Health Institute, Warsaw, Poland (1998-2006). J Antimicrob Chemother 63:269-273.
8. Patzer J, Toleman MA, Deshpande LM, Kaminska W, Dzierzanowska D, Bennett PM, Jones RN, Walsh TR. 2004. *Pseudomonas aeruginosa* strains harbouring an unusual *bla*VIM-4 gene cassette isolated from hospitalized children in Poland (1998-2001). J Antimicrob Chemother 53:451-456.
9. Koh TH, Wang GC, Sng, LH. 2004. IMP-1 and a novel metallo-beta-lactamase, VIM-6, in fluorescent *Pseudomonas* isolated in Singapore. Antimicrob Agents Chemother 48:2334-2336.
10. Crespo MP, Woodford N, Sinclair A, Kaufmann ME, Turton J, Glover J, Velez JD, Castaneda CR, Recalde M, Livermore DM. 2004. Outbreak of carbapenem-resistant *Pseudomonas aeruginosa* producing VIM-8, a novel metallo-beta-lactamase, in a tertiary care center in Cali, Colombia. J Clin Microbiol 42:5094-5101.
11. Pasteran F, Faccone D, Petroni A, Rapoport M, Galas M, Vazquez M, Procopio A. 2005. Novel variant (bla(VIM-11)) of the metallo-beta-lactamase *bla*(VIM) family in a GES-1 extended-spectrum-beta-lactamase-producing *Pseudomonas aeruginosa* clinical isolate in Argentina. Antimicrob Agents Chemother 49:474-475.
12. Juan C, Beceiro A, Gutierrez O, Alberti S, Garau M. 2008. Characterization of the new metallo-beta-lacgtamase VIM-13 and its integron-borne gene from a *Pseudomonas aeruginosa* clinical isolate in Spain. Antimicrob Agent Chemother 52:3589-3596.
13. Schneider I, Keuleyan E, Rasshofer R, Markovska R, Queenan AM, Bauernfeind A. 2008. VIM-15 and VIM-16, two new VIM-2-like metallo-beta-lactamases in *Pseudomonas aeruginosa* isolates from Bulgaria and Germany. Antimicrob Agents Chemother 52:2977-2979.
14. Siarkou VI, Vitti D, Protonotariou E, Ikonomidis A, Sofianou D. 2009. Molecular epidemiology of outbreak-related *Pseudomonas aeruginosa* strains carrying the novel variants blaVIM-17 metallo-beta-lactamase gene. Antimicrob Agents Chemother 53:1325-1330.
15. Montealegre MC, Correa A, Briceno DF, Rosas NC, De La Cadena E, Ruiz SJ, Mojica MF, Camargo RD, Zuluaga I, Marin A, Quinn JP, Villegas MV. 2011. Novel VIM metallo-beta-lactamase variant, VIM-24, from a *Klebsiella pneumoniae* isolate from Colombia. Antimicrob Agents Chemother 55:2428-2430.
16. Papagiannitsis CC, Kotsakis SD, Petinaki E, Vatopoulos AC, Tzelepi E, Miriagou V, Tzouvelekis LS. 2011. Characterization of metallo-beta-lactamase VIM-27, an A57S mutant of VIM-1 associated with *Klebsiella pneumoniae* ST147. Antimicrob Agents Chemother 55:3570-3572.
17. Bogaerts P, Bebrone C, Huang TD, Bouchahrouf W, Degheldre Y, Deplano A, Hoffman K, Glupczynski Y. 2012. Detection and Characterization of VIM-31, a New Variant of VIM-2 with Tyr224His and His252Arg Mutations, in a Clinical Isolate of *Enterobacter cloacae*. Antimicrob Agents Chemother 56:3283-3287.
18. Rodrigues C, Novais A, Machado E, Peixe L. 2014. Detection of VIM-34, a novel VIM-1 variant identified in the intercontinental ST15 *Klebsiella pneumoniae* clone. J Antimicrob Chemother 69:274-275.
19. Iraz M, Duzgun AO, Cicek AC, Bonnin RA, Ceylan A, Saral A, Nordmann P, Sandalli C. 2014. Characterization of novel VIM carbapenemase, VIM-38, and first detection of GES-5 carbapenem-hydrolyzing beta-lactamases in *Pseudomonas aeruginosa* in Turkey. Diagn Microbiol Infect Dis 78:292-294.
20. Papagiannitsis CC, Izdebski R, Baraniak A, Fiett J, Herda M, Hrabak J, Derde LP, Bonten MJ, Carmeli Y, Goossens H, Hryniewick W, Brun-Buisson C, Gniadkowski M. 2015. Survey of metallo-beta-lactamase-producing *Enterobacteriaceae* colonizing patients in European ICUs and rehabilitation units, 2008-11. J Antimicrob Chemother (In press).
