# Supplementary material for: Ceftazidime Is the Key Diversification and Selection Driver of VIM-Type Carbapenemases
Source: mBio. 2018 May 8;9(3):e02109-17. doi: 10.1128/mBio.02109-17 (PMC5941070; doi:10.1128/mBio.02109-17)
Supplement: TABLE S1 [file mbo002183862st1.docx]

**Table S1. Description of the positions predicted to be under selection pressure using the BEAST v1.8 evolutionary program.**

| Evidence | Site | Mean | CPD interval ^a^ | VIM variants | Number of VIM ^b^ |
| --- | --- | --- | --- | --- | --- |
| High | **R228SL** | 6.04 | 3.00-10.94 | **VIM-23/-24/-50**; **VIM-1** | 4 |
|  | **H224LY** | 5.97 | 2.43-12.89 | **VIM-31**; **VIM-26/-28** | 3 |
|  | **Q59R** | 5.65 | 2.84-9.94 | **VIM-36/-3/-6** | 3 |
|  | **A57S** | 5.62 | 2.89-9.71 | **VIM-27/-37**/-49 | 3 |
| Moderate | I14M | 4.13 | 1.95-7.73 | VIM-17 | 1 |
|  | **N165S** | 3.45 | 1.73-6.61 | **VIM-11/**-3**/-6/-50**; **VIM-54** | 5 |
|  | S35N | 3.38 | 1.74-6.32 | VIM-30 | 1 |
|  | M10L | 3.37 | 1.68-6.41 | VIM-38 | 1 |
|  | V246I | 3.34 | 1.69-6.37 | VIM-42 | 1 |
|  | E26D | 3.30 | 1.64-6.24 | VIM-43 | 1 |
|  | **N215SK** | 3.14 | 1.46-5.84 | **VIM-19/-29** | 2 |
|  | T142AV | 3.13 | 1.68-5.38 | VIM-8/-9 | 2 |
|  | A146VT | 3.13 | 1.67-5.38 | VIM-14; VIM-40 | 2 |
|  | T33AI | 3.11 | 1.66-5.34 | VIM-45; VIM-39 | 2 |
|  | F258Y | 2.31 | 1.05-4.45 | VIM-10 | 1 |
|  | **H252R** | 2.29 | 1.13-4.27 | **VIM-20**; **VIM-29** | 2 |
| Low | **Y218F** | 2.09 | 1.06-3.44 | **VIM-15**; **VIM-33** | 2 |

BEAST v1.8 program was used to predict the positions under positive selection ( ω >1) using Robust Counting. The amino acid changes introduced by the site-directed mutagenesis and the *bla*VIM variants constructed are marked in bold. ^a^ Positions are classified as high, moderate and low evidence according to the cumulative posterior density (CPD) intervals, corresponding to 99%, 95% and 90% intervals, respectively.

^b^ Number of VIM refers to the number of VIM enzymes with the corresponding mutation.
