# Supplementary material for: Ceftazidime Is the Key Diversification and Selection Driver of VIM-Type Carbapenemases
Source: mBio. 2018 May 8;9(3):e02109-17. doi: 10.1128/mBio.02109-17 (PMC5941070; doi:10.1128/mBio.02109-17)
Supplement: TABLE S2 [file mbo002183862st2.docx]

**Table S2A. VIM variants constructed by site-directed mutagenesis in VIM-2 cluster.**

|  | VIM  variants ^a^ | Site-specific changes ^b^ | | | | | | |
| --- | --- | --- | --- | --- | --- | --- | --- | --- |
|  |  | **High evidence** | | | | **Moderate evidence** | | **Low evidence** |
|  |  | **Q59R** | **Y224H** | **R228S** | **R228L** | **N165S** | **H252R** | **Y218F** |
|  | VIM-2 |  |  |  |  |  |  |  |
| Single  mutants | VIM-36 | X |  |  |  |  |  |  |
|  | VIM-2_Y224H_ |  | X |  |  |  |  |  |
|  | VIM-23 |  |  | X |  |  |  |  |
|  | VIM-24 |  |  |  | X |  |  |  |
|  | VIM-11 |  |  |  |  | X |  |  |
|  | VIM-20 |  |  |  |  |  | X |  |
|  | VIM-15 |  |  |  |  |  |  | X |
| Double mutants | VIM-2_Q59R+R228S_ | X |  | X |  |  |  |  |
|  | VIM-2_Q59R+R228L_ | X |  |  | X |  |  |  |
|  | VIM-6 | X |  |  |  | X |  |  |
|  | VIM-2_Q59R+H252R_ | X |  |  |  |  | X |  |
|  | VIM-2_Q59R+Y218F_ | X |  |  |  |  |  | X |
|  | VIM-31 |  | X |  |  |  | X |  |
|  | VIM-2_R228S+H252R_ |  |  | X |  |  | X |  |
|  | VIM-2_R228S+Y218F_ |  |  | X |  |  |  | X |
|  | VIM-2_R228L+H252R_ |  |  |  | X |  | X |  |
|  | VIM-2_R228L+Y218F_ |  |  |  | X |  |  | X |
|  | VIM-50 |  |  |  | X | X |  |  |
|  | VIM-2_H252R+Y218F_ |  |  |  |  |  | X | X |
| Triple mutants | VIM-2_R228S+H252R+Y218F_ |  |  | X |  |  | X | X |
|  | VIM-2_R228L+H252R+Y218F_ |  |  |  | X |  | X | X |
| ^a^ VIM variants constructed from *bla*VIM-2 divided into single, double and triple variants. Those VIM variants described in the nature are named with the GenBank original name.  ^b^ Amino acid changes corresponding to non-synonymous mutations introduced by site-directed mutagenesis in *bla*VIM-2 divided into three categories (high, moderate and low evidence) based on the prediction made using the BEAST v1.8 evolutionary program. The symbol "X" indicates presence. | | | | | | | | |

**Table S2B. VIM variants constructed by site-directed mutagenesis in VIM-4 cluster.**

|  | **VIM**  **variants ^a^** | **Site-specific changes ^b^** | | | | | | |
| --- | --- | --- | --- | --- | --- | --- | --- | --- |
|  |  | **High evidence** | | | **Moderate evidence** | | | **Low evidence** |
|  |  | **A57S** | **H224L** | **R228S** | **N165S** | **N215K** | **H252R** | **Y218F** |
|  | VIM-4 |  |  |  |  |  |  |  |
| **Single**  **mutants** | VIM-37 | X |  |  |  |  |  |  |
|  | VIM-28 |  | X |  |  |  |  |  |
|  | VIM-1 |  |  | X |  |  |  |  |
|  | VIM-54 |  |  |  | X |  |  |  |
|  | VIM-19 |  |  |  |  | X |  |  |
|  | VIM-4_H252R_ |  |  |  |  |  | X |  |
|  | VIM-4_Y218F_ |  |  |  |  |  |  | X |
| **Double mutants** | VIM-4_A57S+H224L_ | X | X |  |  |  |  |  |
|  | VIM-27 | X |  | X |  |  |  |  |
|  | VIM-4_A57S+N215K_ | X |  |  |  | X |  |  |
|  | VIM-4_A57S+H252R_ | X |  |  |  |  | X |  |
|  | VIM-4_A57S+Y218F_ | X |  |  |  |  |  | X |
|  | VIM-26 |  | X | X |  |  |  |  |
|  | VIM-4_H224L+N215K_ |  | X |  |  | X |  |  |
|  | VIM-4_H224L+H252R_ |  | X |  |  |  | X |  |
|  | VIM-4_H224L+Y218F_ |  | X |  |  |  |  | X |
|  | VIM-4_R228S+N215K_ |  |  | X |  | X |  |  |
|  | VIM-4_R228S+H252R_ |  |  | X |  |  | X |  |
|  | VIM-33 |  |  | X |  |  |  | X |
|  | VIM-4_N215K+H252R_ |  |  |  |  | X | X |  |
|  | VIM-4_N215K+Y218F_ |  |  |  |  | X |  | X |
|  | VIM-4_H252R+Y218F_ |  |  |  |  |  | X | X |
| **Triple mutants** | VIM-29 |  |  | X |  | X | X |  |
| **^a^** VIM variants constructed from *bla*VIM-4 divided into single, double and triple variants. Those VIM variants described in the nature are named with the GenBank original name.  **^b^** Amino acid changes corresponding to non-synonymous mutations introduced by site-directed mutagenesis in *bla*VIM-4 divided into three categories (high, moderate and low evidence) based on the prediction made using the BEAST v1.8 evolutionary program. The symbol "X" indicates presence. | | | | | | | | |
