# Supplementary material for: Ceftazidime Is the Key Diversification and Selection Driver of VIM-Type Carbapenemases
Source: mBio. 2018 May 8;9(3):e02109-17. doi: 10.1128/mBio.02109-17 (PMC5941070; doi:10.1128/mBio.02109-17)
Supplement: TABLE S3 [file mbo002183862st3.docx]

**Table S3. Mutations obtained in the different serial passages experiments.**

|  | **VIM-2** | | | **VIM-4** | | |
| --- | --- | --- | --- | --- | --- | --- |
|  | **High evidence** | **Moderate evidence** | **Low evidence** | **High evidence** | **Moderate evidence** | **Low evidence** |
| **Increasing concentration of CAZ** | R228L  (9%) | N165S  (27.3%) | - | R228S  (9%) | N165S  (18.2%) | E30A  (9%) |
|  | Q59R  (9%) |  |  | Q59R  (9%) |  |  |
| **Increasing concentration of IMI/MER/ERT** | - | - | - | - | - | - |
| **Increasing concentration of CAZ and a fixed subinhibitory IMI concentration** | Q59R  (9%) | N165S  (18.2%) | - | Q59R **^a^**  (9%) | N165S  (27.3%) | - |
| **Increasing concentration of CAZ and IMI in alternative days** | Q59R  (9%) | N165S  (27.3%) | - | R228S  (9%) | N165S  (27.3%) | - |
| **Increasing concentration of CTX** | - | - | - | - | - | - |
|  |  |  |  |  |  |  |

The mutations found are classified in high evidence (99% cumulative posterior density interval that exclude ω =1), moderate evidence (95% cumulative posterior density interval that exclude ω=1) and low evidence (90% cumulative posterior density interval that exclude ω =1) to be under selection according to the Bayesian prediction made using the BEAST v1.8 evolutionary program. The percentage indicated for each mutation refers to the number of lineages that had the change with respect to the total of lineages involved in the experiment.

**^a^** The mutation Q59R at increasing concentrations of CAZ and a fixed concentration of IMI in VIM-4 was selected in combination with N165S (non-described double mutant).

CAZ, ceftazidime; IMI, imipenem; MER, meropenem; ERT, ertapenem; CTX, cefotaxime.
