# Supplementary material for: Ceftazidime Is the Key Diversification and Selection Driver of VIM-Type Carbapenemases
Source: mBio. 2018 May 8;9(3):e02109-17. doi: 10.1128/mBio.02109-17 (PMC5941070; doi:10.1128/mBio.02109-17)
Supplement: TEXT S2 [file mbo002183862s2.docx]

**Text S2. Cloning and transformation.**

The original *Pseudomonas aeruginosa* strains carrying the *bla*_VIM-2_ and *bla*_VIM-4_ genes were requested to the investigators who described them the first time (1,2). The genes amplification was carried out using the primers VIM2-F (5´-CCATGGCTTTGGCTCCACTA-3´) and VIM2-R (5´-GCATCTGCCTGCTACTCAA) for *bla*_VIM-2_ and PoiVIM1-F (5´-TATGCCGCACCCACCCCTATG-3´) and PoiVIM1-R (5´-CTGCTACTCGGCGACTGAGC-3´) for *bla*_VIM-4_ (3).

Once the genes were cloned in pCR^TM^-Blunt II-TOPO and transformed into *Escherichia coli* TOP10, the nucleotide sequence was confirmed by sequencing analysis and the correct direction of the insert was verified by PCR using the universal primers M13-F (5´-GTAAAACGACGGCCAG-3´) and M13-R (5´-CAGGAAACAGCTATGAC-3´).

**References**

1. Poirel L, Naas T, Nicolas D, Collet L, Bellais S, Cavallo JD, Nordmann P.2000. Characterization of VIM-2, a carbapenem-hydrolyzing metallo-beta-lactamase and its plasmid- and integron-borne gene from a *Pseudomonas aeruginosa* clinical isolate in France. Antimicrob Agents Chemother 44:891-897.
2. [Patzer J](https://www.ncbi.nlm.nih.gov/pubmed/?term=Patzer%20J%5BAuthor%5D&cauthor=true&cauthor_uid=14749341), [Toleman MA](https://www.ncbi.nlm.nih.gov/pubmed/?term=Toleman%20MA%5BAuthor%5D&cauthor=true&cauthor_uid=14749341), [Deshpande LM](https://www.ncbi.nlm.nih.gov/pubmed/?term=Deshpande%20LM%5BAuthor%5D&cauthor=true&cauthor_uid=14749341), [Kamińska W](https://www.ncbi.nlm.nih.gov/pubmed/?term=Kami%C5%84ska%20W%5BAuthor%5D&cauthor=true&cauthor_uid=14749341), [Dzierzanowska D](https://www.ncbi.nlm.nih.gov/pubmed/?term=Dzierzanowska%20D%5BAuthor%5D&cauthor=true&cauthor_uid=14749341), [Bennett PM](https://www.ncbi.nlm.nih.gov/pubmed/?term=Bennett%20PM%5BAuthor%5D&cauthor=true&cauthor_uid=14749341), [Jones RN](https://www.ncbi.nlm.nih.gov/pubmed/?term=Jones%20RN%5BAuthor%5D&cauthor=true&cauthor_uid=14749341), [Walsh TR](https://www.ncbi.nlm.nih.gov/pubmed/?term=Walsh%20TR%5BAuthor%5D&cauthor=true&cauthor_uid=14749341). 2004. *Pseudomonas aeruginosa* strains harbouring an unusual *bla*VIM-4 gene cassette isolated from hospitalized children in Poland (1998-2001). J Antimicrob Chemother 53:451-456.
3. Rodríguez-Martínez J, Nordmann P, Fortineau N, Poirel L. 2009. VIM-19, a metallo-ß-lactamase with increased carbapenemases activity from *Escherichia coli* and *Klebsiella pneumoniae*. Antimicrob Agents Chemother 54:471-476.
