# Supplementary material for: Ceftazidime Is the Key Diversification and Selection Driver of VIM-Type Carbapenemases
Source: mBio. 2018 May 8;9(3):e02109-17. doi: 10.1128/mBio.02109-17 (PMC5941070; doi:10.1128/mBio.02109-17)
Supplement: TEXT S3 [file mbo002183862s3.docx]

**Text S3. Site-directed mutagenesis protocol.**

The primers used for construction of the VIM variants are shown in Table S4. Mutagenesis PCR conditions were 2 minutes at 95ºC, 18 cycles of 1 minute at 35ºC, 1 minute at 60ºC and 6 minutes 30 seconds at 68ºC, with a final extension at 68ºC for 15 minutes. The PCR protocol was followed by a *Dpn*I digestion at 37ºC for 1 hour. All the mutants constructed are summarised in Table S2.
