# Supplementary material for: Ceftazidime Is the Key Diversification and Selection Driver of VIM-Type Carbapenemases
Source: mBio. 2018 May 8;9(3):e02109-17. doi: 10.1128/mBio.02109-17 (PMC5941070; doi:10.1128/mBio.02109-17)
Supplement: TABLE S4 [file mbo002183862st4.docx]

**Table S4. Primers used for the mutagenesis of *bla*_VIM-2_ and *bla*_VIM-4_.**

|  | Primer name | Sequence (5’-3’) |
| --- | --- | --- |
| VIM-2 cluster | Mut2-59R-F | GGTCGCATATCGCAACGCGGTCGTTTGATGGCGCAGTC |
|  | Mut2-59R-R | GACTGCGCCATCAAACGACCGCGTTGCGATATGCGACC |
|  | Mut2-218F | TACGTCCCGTCTGCGAGTGTGCTCTTTGGTGGTTGTGCGATT |
|  | Mut2-218R | AATCGCACAACCACCAAAGAGCACACTCGCAGACGGGACGTA |
|  | Mut2-224F | GGTGGTTGTGCGATTCATGAGTTGTCACGCACGTCTGCGG |
|  | Mut2-224R | CCGCAGACGTGCGTGACAACTCATGAATCGCACAACCACC |
|  | Mut2-228S-F | GCGATTTATGAGTTGTCAAGCACGTCTGCGGGGAACGTGG |
|  | Mut2-228S-R | CCACGTTCCCCGCAGACGTGCTTGACAACTCATAAATCGC |
|  | Mut2-228L-F | gcgatttatgagttgtcactcacgtctgcggggaacgtgg |
|  | Mut2-228L-R | CCACGTTCCCCGCAGACGTGAGTGACAACTCATAAATCGC |
|  | Mut2-252F | cattgagcggattcaacaacgctacccggaagcacagttc |
|  | Mut2-252R | GAACTGTGCTTCCGGGTAGCGTTGTTGAATCCGCTCAATG |
| VIM-4 cluster | Mut4-57F | GGTGTTTGGTCGCATATCTCAACGCAGTCGTTTGATGGCG |
|  | Mut4-57R | CGCCATCAAACGACTGCGTTGAGATATGCGACCAAACACC |
|  | Mut4-215F | GTATACGTCCCGTCAGCGAAAGTGCTATACGGTGGTTGTG |
|  | Mut4-215R | CACAACCACCGTATAGCACTTTCGCTGACGGGACGTATAC |
|  | Mut4-218F | CCGTCAGCGAACGTGCTATTCGGTGGTTGTGCCGTTCATG |
|  | Mut4-218R | CATGAACGGCACAACCACCGAATAGCACGTTCGCTGACGG |
|  | Mut19-218F | CCGTCAGCGAAAGTGCTATTCGGTGGTTGTGCCGTTC |
|  | Mut19-218R | GAACGGCACAACCACCGAATAGCACTTTCGCTGACGG |
|  | Mut4-224F | CGGTGGTTGTGCCGTTCTTGAGTTGTCACGCACGTCTGCG |
|  | Mut4-224R | CGCAGACGTGCGTGACAACTCAAGAACGGCACAACCACCG |
|  | Mut4-228F | CCGTTCATGAGTTGTCAAGCACGTCTGCGGGGAACGTG |
|  | Mut4-228R | CACGTTCCCCGCAGACGTGCTTGACAACTCATGAACGG |
|  | Mut4-252F | CGTTGAGCGGATTCAAAAACGCTACCCGGAAGCAGAGGTC |
|  | Mut4-252R | GACCTCTGCTTCCGGGTAGCGTTTTTGAATCCGCTCAACG |
|  | | |
