# Supplementary material for: Ceftazidime Is the Key Diversification and Selection Driver of VIM-Type Carbapenemases
Source: mBio. 2018 May 8;9(3):e02109-17. doi: 10.1128/mBio.02109-17 (PMC5941070; doi:10.1128/mBio.02109-17)
Supplement: TEXT S4 [file mbo002183862s4.docx]

**Text S4. Experimental evolution details.**

To carry out the experimental evolution assays, the *E. coli* XL1-red strains harboring the *bla*_VIM_ genes encoding for VIM-4, VIM-1 and VIM-2 were submitted to serial passages in presence of increasing concentrations of beta-lactam antibiotics. Before starting, the original MIC values were determined for all beta-lactams used in the experimental evolution assays (CAZ, CTX, IMP, MER and ERT). Briefly, for each assay, 2 µl of an overnight Luria Broth culture were inoculated in 11 independent tubes containing fresh media at 50 μg/ml of kanamycin and 250 μg/ml of cloxacillin. The initial concentration of each selector antibiotic in those 11 independent experiments was 2-fold lower than the original MIC. After 24 hours under agitation (250 rpm) at 37ºC, 2 µl of the culture were inoculated in 2 ml of fresh media (dilution 1:1000) containing two times the previous antibiotic concentration until growth was not detected. The highest concentrations reached were from 256 µg/ml (for *bla*_VIM-4_ and *bla*_VIM-2_) to 1024 µg/ml (for *bla*_VIM-1_) of CAZ, or 256 µg/ml of CTX. Among the carbapenems tested (IMI, MER and ERT), the highest concentration with bacterial growth was 64 µg/ml, with the exception of VIM-2 at increasing concentrations of IMI, where the highest concentration with growth was 128 µg/ml. There are high possibilities of mutations in other genes contributing to the increase in the MIC, like hyperproduction of chromosomal AmpC or loss of porins but the aim of this experiment is to analyze exclusively the mutations occurred in the sequence of *bla*_VIM_ genes. Plasmid extraction was performed from each evolution experiment grown at the maximum concentration of each antibiotic using the QIAprep Spin Miniprep Kit (Qiagen, Düsseldorf, Germany). The plasmid extracted was transformed into *E. coli* XL1-red using 50 μg/ml of kanamycin and 2-fold higher than original MIC for selection. For each evolution experiment, between 2-10 clones were picked up for a new plasmid extraction and a new transformation using 50 μg/ml of kanamycin. A screening with commercial disks containing different beta-lactam antibiotics was performed in order to identify patterns of reduced susceptibility. All clones that yielded different susceptibility profiles than the ancestor were selected. The plasmid DNA of each clone selected was reextracted and analyzed by sequencing. The plasmids containing mutations in the *bla*_VIM_ genes were transformed into *E. coli* TOP10 to check the MICs. The MIC values were determined at least three times.

This strategy has two main limitations. One of them is that a resistant phenotype can be conferred by commonly encountered mutations in genes other than *bla*VIM. As a result, resistant isolates carrying mutations in the *bla*VIM gene are recovered with low frequency. 
Secondly, the possibility of recovering a high diversity of mutations or double mutants in *bla*VIM is decreased for two reasons: a) in a non-compartmentalized culture, selection will favor the emergence of a single type of mutant and b) only a small fraction of cells is transferred from one tube to the next during serial passage, resulting in population bottlenecks that further reduce the probability of detecting *bla*VIM mutants present in low copy.
